# On the Hardening and Softening Behaviors of Additively Manufactured and Forged Inconel 718 Alloys Under Non-Isothermal Heat Treatments

**DOI:** 10.3390/ma18225174

**Published:** 2025-11-14

**Authors:** Yufeng Dong, Yetao Cheng, Jie Tang, Yubin Ke, Jie Teng, Fulin Jiang

**Affiliations:** 1 State Key Laboratory of Cemented Carbide, College of Materials Science and Engineering, Hunan University, Changsha 410082, China; yufengdong1027@hnu.edu.cn (Y.D.); chengyt1028360301@163.com (Y.C.); tengjie@hnu.edu.cn (J.T.); 2 Beihai Petrochemical and New Materials Industry Development Promotion Center, Beihai 536000, China; 3 College of Materials Science and Engineering, Central South University, Changsha 410083, China; 4 Spallation Neutron Source Science Center, Dongguan 523803, China; 5 Institute of High Energy Physics, Chinese Academy of Sciences, Beijing 100049, China

**Keywords:** Inconel 718 alloy, additive manufacture, non-isothermal heat treatment, microstructure, hardness

## Abstract

During the heat treatment of nickel-based superalloy (for instance Inconel 718 alloy), the non-isothermal heating and cooling processes significantly influenced precipitation behaviors as well as the final mechanical properties. This study compared the precipitation behaviors and the resulting hardening and softening behaviors of additively manufactured and conventionally forged Inconel 718 alloys under non-isothermal heat treatment processes. The results indicated that additively manufactured Inconel 718 alloy accelerated aging precipitation behavior due to the fine dendritic structure during both heating and cooling processes. As a result, the additively manufactured alloy reached peak hardness of ~480 HV at ~650 °C (~100 °C earlier than the forged alloy’s peak hardness of ~460 HV at ~750 °C) during heating and gained almost constant hardness during cooling. Further, the heating rate significantly affected the precipitation behaviors of γ″ and γ′ phases in both alloys. Slower heating rates provided sufficient time for phase transformation, leading to a more pronounced precipitation., e.g., the volume fraction of precipitates in the SLM alloy increased from 1.5% at 5 °C/min to 5.9% at 0.5 °C/min when heated to 850 °C. During cooling process, the twisted grain boundaries of additively manufactured alloy facilitated the precipitation of δ-phase, which in turn inhibited the formation of γ/γ′/γ″ phase. Both alloys exhibited minimum hardness of ~380–390 HV at 1000 °C due to complete dissolution of strengthening phases. This study provides a comparative understanding of non-isothermal phase evolution in AM and forged Inconel 718, which is critical for optimizing heat treatment in aerospace applications.

## 1. Introduction

Inconel 718 alloy is widely used in important components such as aero engines due to its excellent high-temperature oxidation resistance and comprehensive mechanical properties across a wide range of temperatures [1]. As material performance requirements increase, traditional manufacturing methods are no longer sufficient, leading to higher demands for lightweight, complex, and integrated nickel-based high-temperature alloys. Additive manufacturing (AM) technology has emerged as a solution, offering unique advantages such as “from fragmentation to accumulation,” “top-down,” and “from scratch” fabrication, along with high efficiency [2,3]. The combination of its superior high-temperature strength, good weldability, and moderate cost makes IN718 particularly suitable for the AM process [4]. Among AM technologies, selective laser melting (SLM) has revolutionized metal processing, enabling the production of complex geometries with high precision, minimal segregation, and no need for molds [2,5]. Consequently, extensive research has been conducted on SLM-processed Inconel 718 alloys [6,7,8,9,10,11,12,13,14,15,16,17,18].

However, the intrinsic characteristics of the SLM process, characterized by extreme thermal cycles involving rapid melting and solidification, inevitably introduce microstructural heterogeneities such as columnar grain structures, micro-segregation, and the precipitation of undesirable non-equilibrium phases (e.g., Laves phase). These defects can lead to anisotropic mechanical properties and a significant degradation in performance, thereby necessitating post-process heat treatments to achieve a homogeneous microstructure and enhance mechanical properties [4].

The mechanical properties of Inconel 718 are predominantly governed by the precipitation of strengthening phases within its face-centered cubic (FCC) γ matrix. The primary strengthening phase is the meta-stable body-centered tetragonal (BCT) γ″ phase (Ni_3_Nb), which induces significant coherency strain hardening due to its large lattice mismatch with the matrix. The auxiliary strengthening phase is the stable face-centered cubic (FCC) γ′ phase (Ni_3_(Al,Ti)), which exhibits excellent thermal stability. Upon prolonged exposure to temperatures above approximately 650 °C, the γ″ phase tends to transform into the stable orthorhombic δ phase (Ni_3_Nb), which typically precipitates at grain boundaries. While a controlled amount of δ phase can benefit grain refinement and stress relief, excessive precipitation depletes the strengthening element Nb and reduces the volume fraction of γ″, thereby deteriorating mechanical strength [19]. Additionally, the brittle Laves phase (e.g., Ni,Fe,Cr)_2_(Nb,Mo,Ti) formed in the last solidifying liquid pools during solidification is generally considered detrimental, as it consumes Nb and promotes crack initiation [20]. The formation, dissolution, and transformation of these phases are critically influenced by the thermal history. Therefore, understanding the non-isothermal precipitation behavior of these phases is fundamental to tailoring heat treatments for optimal performance.

Solution treatment aims to dissolve the Laves phase into the matrix, resulting in a single-phase austenite (γ) structure, which is essential for subsequent precipitation of strengthening phases. Aging treatment is crucial to fully precipitate these phases and enhance mechanical properties [15,21,22]. Previous studies have focused on the heat treatment of SLM-processed Inconel 718, examining its effects on microstructure and properties [9,10,14,15,17,18]. Due to the unique characteristics of the SLM process, the microstructure of SLM-processed Inconel 718 differs significantly from that of traditional cast and forged materials [4,6,23,24,25]. For instance, Huang et al. [20] found that SLM-processed Inconel 718 requires higher solution treatment temperatures (1080 °C) to achieve a homogeneous microstructure. The cooling rate after solution treatment significantly affects the characteristics of precipitated phases (size, quantity, etc.), which in turn influence mechanical properties. Compared to traditional forging, SLM-processed alloys exhibit earlier peak aging times; Tabaie et al. [26] found that the evolution of the γ″ and δ phases of SLMed-IN718 was different from that of conventional forged materials at high heating rates, and the dissolution of the γ″ and δ phases was delayed and moved to higher temperatures.

Non-isothermal heat treatment, involving heating, holding, and cooling processes, has received less attention compared to isothermal heat treatment. The intrinsic mechanisms governing the precipitation behavior of nickel-based high-temperature alloys prepared by additive manufacturing and traditional forging remain poorly understood [20,26,27,28,29]. This study systematically investigates the effects of cooling and heating rates on the precipitation behavior of additively manufactured and conventionally forged Inconel 718 alloys by utilizing hardness test, DSC, SEM, EBSD and TEM characterization, The related hardening and softening behaviors of both alloys during non-isothermal heat treatment processes were also compared.

## 2. Materials and Methods

### 2.1. Materials

The employed Inconel 718 alloys in this study are both forged (DF) and selective laser melting (SLM) additively manufactured states. The elemental compositions of the alloys are shown in Table 1. In the additive manufacturing process, Inconel 718 alloy powder with an average particle size of 15–53 μm was used by gas atomization, and the elemental composition is shown in Table 1.

### 2.2. Sample Preparation

Inconel 718 alloy samples (76 mm × 11 mm × 9 mm) were prepared using SLM with a strip scanning strategy. The printing parameters included a laser power of 110–174 W, scanning speed of 400–825 mm/s, scanning spacing of 0.08 mm, and powder layer thickness of 30 μm. Argon gas was used to protect the printing process. In the sample coordinate system, the XOY surface is the scanning surface of the laser, which is printed layer by layer upward along the building direction (BD), and the other two sides are the YOZ surface and XOZ surface, respectively.

### 2.3. Microstructure and Property Analysis

**Figure 1:**
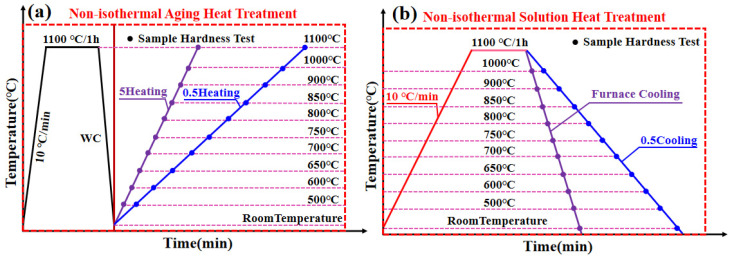
Schematic diagram of non-isothermal heat treatment routes: (**a**) Heating routes and (**b**) colling route after solid solution.

Differential scanning calorimetry (DSC) was performed using a TG-DSC synchronous thermal analyzer (PerkinElmer, Inc, Waltham, MA, USA) with cylindrical samples (Φ3 mm × 2 mm) at a heating rate of 10 °C/min from room temperature to 1400 °C under argon atmosphere (flow rate: 100 mL/min). Based on the DSC results, a new non-isothermal heat treatment process was designed and implemented, as illustrated in Figure 1, which includes both non-isothermal solution heat treatment and non-isothermal aging heat treatment routes.

Following the non-isothermal heat treatments, microstructural characterization was performed. Samples for OM, SEM and EBSD analysis were prepared as cuboid shapes (10 mm × 3 mm × 3 mm), while TEM samples were prepared as thin foils. All samples were cut using wire electrical discharge machining (EDM), followed by ultrasonic cleaning.

For OM and SEM observation, samples were mounted, ground, polished, and etched using a solution of 80 vol.% HCl + 13 vol.% HF + 7 vol.% HNO_3_ (chemical etching time: 100–130 s; electrolytic etching at 5 V for 1–3 s). For EBSD analysis, samples were electrolytically polished in 20 vol.% HClO_4_ + 80 vol.% CH_3_OH at 25 V and −25 °C for 25 s. For TEM observation, thin foils were prepared by twin-jet electropolishing in 7 vol.% HClO_4_ + 90 vol.% CH_3_OH at 25 V and −25 °C for 7–8 min.

The volume fraction of precipitates was quantitatively analyzed from SEM images using ImageJ software (Fiji Is Just ImageJ). The analysis procedure included: (1) applying consistent thresholding to differentiate precipitates from the matrix, (2) converting the images to binary format, and (3) calculating the area fraction of precipitates, which represents the volume fraction. At least five different fields of view were analyzed for each condition to ensure statistical reliability.

Microstructure observations were conducted using a TESCAN field emission scanning electron microscope (SEM) (FEI QUANTA 200 Scanning Electron Microscope, FEI Company, USA) and electron backscatter diffraction (EBSD). SEM observations were conducted using a TESCAN field emission SEM. TEM observations were carried out using an FEI Tecnai G2 F20 (TEM-FEI Tecnai G2 F20, FEI Company, USA) transmission electron microscope operated at 200 kV. Although X-ray diffraction (XRD) is a common technique for phase identification, this study employed TEM and selected area electron diffraction (SAED) for their superior ability to resolve nanoscale precipitates and characterize their crystallographic relationships with the matrix, which was crucial for understanding the precipitation behavior of γ″ and γ′ phases.

Vickers microhardness was measured using a HV-1000BZ (Shanghai Lidun Instrument & Meter Testing Technology Co., Ltd., Shanghai, China) tester with a load of 0.2 kgf and a dwell time of 15 s. At least five measurements were taken per sample and average.

Room temperature tensile tests were conducted using an Instron-3382 (Instron Corporation, Norwood, MA, USA) universal testing machine at a displacement rate of 0.5 mm/min. The tensile specimen geometry had a gauge length of 7 mm.

## 3. Results and Discussion

### 3.1. DSC Analysis

**Figure 2:**
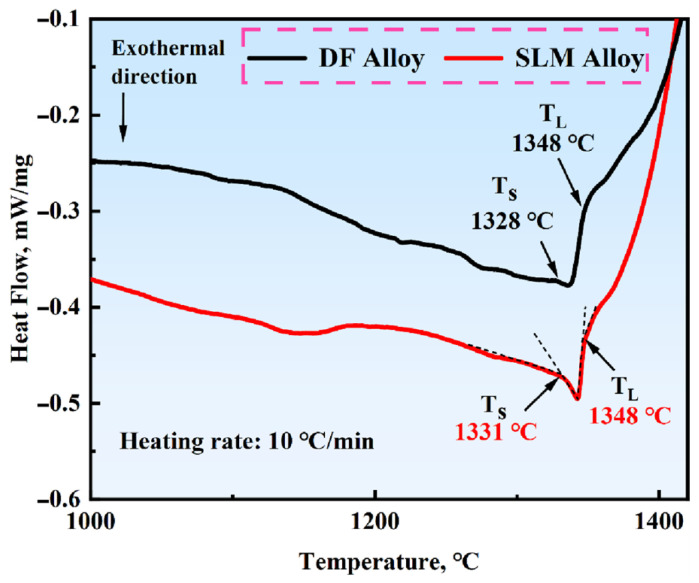
DSC curves of Inconel 718 alloys. (The dashed line in the figure indicates the epitaxial method).

To determine the solidus and liquidus temperatures of the DF and SLM alloys, as well as the temperature at which the δ-phase completely dissolves into the matrix, DSC tests were conducted. Figure 2 shows the DSC curves of Inconel 718 alloys heated from room temperature to 1400 °C, and Table 2 lists the solidus and liquidus temperatures along with the solidification ranges of the alloys. By using the extrapolation method to determine the solidus and liquidus temperatures, it can be observed that the solidus and liquidus temperatures of the two alloys are close to each other. The wider the temperature range of the alloy, the larger the solid–liquid two-phase coexistence range, which increases the likelihood of solidification cracks. The relatively sharp inflection point in the DSC curve may be related to the low-segregation characteristics of the SLM alloy, which refers to the uniform distribution of alloying elements resulting from rapid solidification during the SLM process. It is preliminarily concluded that the δ-phase in SLM alloys will dissolve into the matrix at approximately 1120 °C, and further SEM analysis is required to confirm this conclusion.

### 3.2. Initial Microstructure

**Figure 3:**
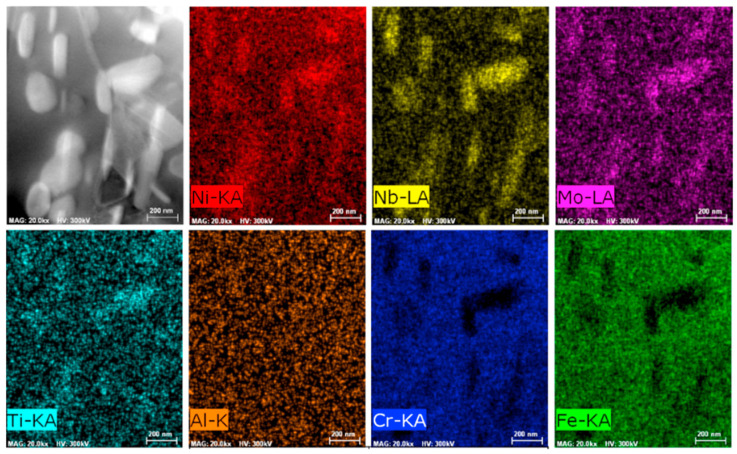
EDS data from the initial microstructure [30].

Figure 3 presents the EDS (Energy Dispersive Spectroscopy) results of the initial microstructure of Inconel 718 alloy. From the EDS mapping and quantitative analysis in the figure, the distribution uniformity of main alloying elements (such as Ni, Cr, Nb, Mo) and the presence of potential elemental segregation in different regions of the initial microstructure can be directly observed. This elemental distribution information provides essential experimental support for subsequent analysis of the initial microstructure formation mechanism, as well as the correlation between microstructure evolution (under subsequent heat treatment) and mechanical properties.

**Figure 4:**
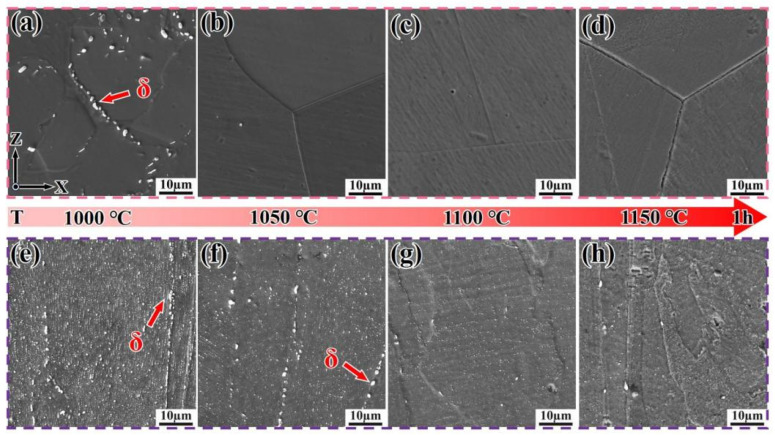
Microstructure evolution of Inconel 718 alloys after solution treatment at different temperatures. (**a**–**d**) DF alloy; (**e**–**h**) SLM alloy.

In order to further determine the temperature at which the δ-phase dissolves into the matrix in both DF and SLM alloys, solution treatment was conducted at temperatures ranging from 1000 °C to 1150 °C for 1 h. The evolution of the microstructures is shown in Figure 4. When the solution temperature was 1000 °C, the δ-phase (nanometer-scale precipitate) precipitated at the grain boundaries of both SLM and DF alloys (Figure 4a,b). The volume fraction of the δ-phase in SLM alloys was approximately 3.6%, with smaller sizes and a chain-like distribution along the Z-direction (i.e., the build direction), accompanied by short rods (with an aspect ratio typically ranging from 3 to 5) and granules. In contrast, the δ-phase in DF alloys exhibited a linear distribution along the grain boundaries of the γ-phase (micrometer-scale matrix)—the γ-phase, as the alloy’s matrix, is sufficiently coarse to be clearly observed via SEM (serving as the background in Figure 4), while the δ-phase requires higher-magnification TEM for detailed characterization.” However, when the temperature increased to 1050 °C, the δ-phase in SLM alloys transformed into granular form (Figure 4f), while in DF alloys, the δ-phase was almost completely dissolved into the matrix (Figure 4b). It was only when the temperature reached 1100 °C that the δ-phase in SLM alloys was nearly completely dissolved into the matrix (Figure 4g,h). In summary, the evolution of the δ-phase follows a sequence from long needles to short rods and finally to granules before complete dissolution into the matrix [25].

**Figure 5:**
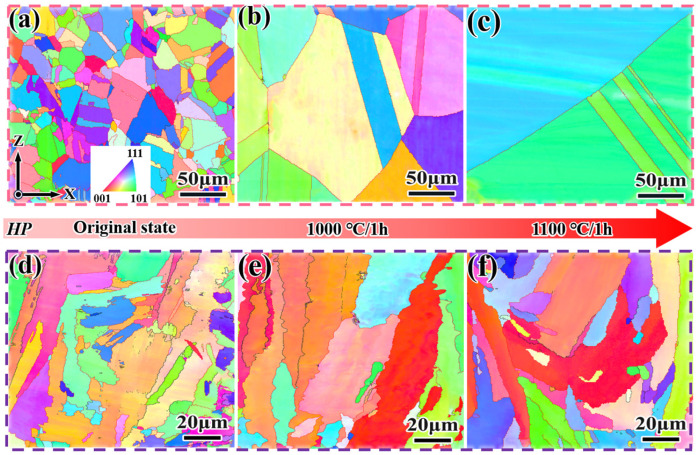
EBSD-IPF (Inverse Pole Figure) maps of Inconel 718 alloys in the as-printed and solution-treated states. (**a**–**c**) DF alloy; (**d**–**f**) SLM alloy.

Figure 5 shows the EBSD-IPF (Inverse Pole Figure) maps of DF and SLM alloys in their as-printed and solution-treated states. The adjacent grains of both alloys are primarily separated by high-angle grain boundaries. From Figure 5a–c, it can be observed that the grains of the as-printed DF alloy are equiaxed, with an average grain size of 8.64 μm, relatively straight grain boundaries, and the presence of obvious twin structures. The grain orientations are dominated by <101> and <111> directions. After solution treatment at 1000 °C for 1 h, significant grain growth occurred. However, when the temperature increased to 1100 °C, the grain size showed a decreasing trend.

From Figure 5d–f, it can be seen that the as-printed SLM alloy exhibits twisted dendrites. This is due to the significant temperature gradient between the substrate and the pre-printed powder material during the SLM process, where heat dissipates in the direction of the substrate (i.e., the Z-direction). This leads to the epitaxial growth of grains, and the extremely fast cooling rate results in the formation of fine-grained structures [29], with an average grain size of 4.85 μm. The grain orientation was dominated by the <001> direction. After solution treatment at 1000 °C for 1 h, the grain orientation was dominated by <001> and <111> directions, with the <001> orientation weakened. The grain size increased only slightly (~4.29 μm), and the distribution of grain sizes became more homogeneous when the temperature was increased to 1100 °C.

**Figure 6:**
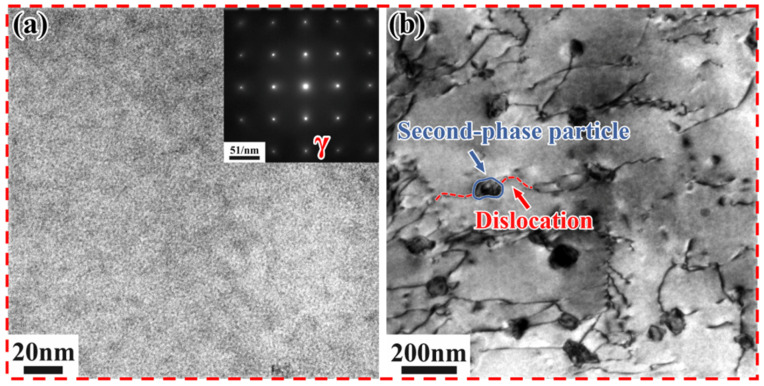
TEM images of the SLM alloy after solution treatment at 1100 °C for 1 h. (**a**) Bright-field image; (**b**) Selected area electron diffraction (SAED) pattern along the [001] crystallographic zone axis.

Figure 6 shows the TEM images of the SLM alloy after solid solution at 1100 °C for 1 h, along with the selected area electron diffraction (SAED) pattern in the upper right corner (along the [001] crystallographic zone axis). In the TEM bright field image and the corresponding SAED pattern, it can be observed that only the matrix γ-phase remains (Figure 6a), confirming that the δ-phase has almost completely dissolved into the matrix. Dislocations are found to bypass the precipitates within the subcrystals (Figure 6b), exhibiting bowed configurations around the obstacles, which hinders dislocation movement and thereby strengthens the matrix [31].

### 3.3. Effect of Heating/Cooing Rates on Hardness

This study employs microhardness as a highly effective and sensitive indicator for tracking precipitation evolution and related strengthening/softening phenomena during non-isothermal treatments. The primary focus here is to establish the correlation between microstructural changes and the corresponding hardening/softening responses. Tensile testing, which provides complementary mechanical property data, will be conducted in a subsequent study focused on overall mechanical performance.

**Figure 7:**
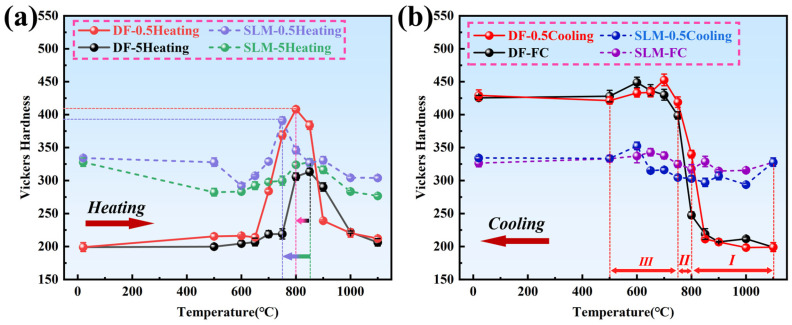
Unified hardness profiles under different thermal conditions. (The arrow indicates the time offset at which the hardness peak is reached.) (**a**) Heating processes (5 °C/min vs. 0.5 °C/min); (**b**) Cooling processes (FC vs. 0.5 °C/min).

Figure 7a shows the hardness variation curves of Inconel 718 alloys under different heating rates. As can be seen from the figure, the hardness of both alloy samples initially increases and then decreases with increasing temperature. The peak hardness temperature of the DF alloy is advanced by approximately 50 °C as the heating rate slows down, while the peak hardness temperature of the SLM alloy is significantly advanced by approximately 100 °C. This notable acceleration in the aging precipitation process observed in the SLM alloy is consistent with the findings of Tabaie et al. [26], who also reported that SLMed IN718 reaches peak aging faster than its wrought counterpart. This phenomenon is generally attributed to the unique microstructure of the SLM alloy [6,7]. The fine dendritic structure and high dislocation density resulting from rapid solidification provide a significantly higher number of nucleation sites for γ″ and γ′ precipitates, thereby shortening the incubation time and shifting the peak hardness to a lower temperature [17,26]. This indicates that the faster the heating rate, the higher the peak temperature, indirectly suggesting that the SLM alloy accelerates the aging precipitation process and shortens the time required to reach peak hardness. This is because, during the non-isothermal aging process, there are two variables: temperature and time. Important precipitation phases (e.g., γ″ phase and γ′ phase) need to satisfy both conditions simultaneously; otherwise, they cannot precipitate at the peak temperature, leading to a shift in the precipitation temperature range to higher temperatures. Since the γ″ phase is a metastable phase, it undergoes aggregation and growth above 650 °C, eventually transforming into the δ phase [32]. The temperature at which the γ″ phase begins to dissolve and the γ′ phase is completely dissolved is approximately 840 °C. Coupled with the slow heating rate, which provides sufficient time for phase transformation, this results in a sudden drop in the hardness of both alloys within this temperature range.

Figure 7b shows the hardness variation curves of Inconel 718 alloys under different cooling rates. From the figure, it can be observed that both cooling curves of the DF alloy initially show a gradual and rapid increase in hardness, followed by a slower increase. Due to the fast cooling rate (~13 °C/min) during furnace cooling (FC) in the temperature range of 1000 °C to 800 °C (Stage I), the precipitated phases do not have sufficient time to form, resulting in only a small change in hardness. In contrast, the 0.5 °C/min cooling curve has a slower cooling rate in this region, allowing a certain amount of δ-phase to precipitate. The δ-phase precipitates most rapidly at around 940 °C, causing its hardness curve to rise above the FC curve. In the temperature range of 800 °C to 700 °C (Stage II), a sudden increase in hardness occurs in both cooling curves, which may be due to the consumption of excessive Nb (as demonstrated in subsequent sections). However, in the temperature range of 700 °C to 500 °C (Stage III), the FC curve lies above the 0.5 °C/min cooling curve. This is because the cooling rate of FC becomes slower (~4 °C/min) in Stages II and III, providing sufficient time for the δ-phase (Ni_3_Nb) to precipitate and retaining the strengthening phase component Nb. The precipitation of the strengthening phases during 0.5 °C/min cooling is affected by the δ-phase precipitation in Stages I and II, resulting in a reduced amount of γ″ and γ′ phase precipitation in Stage III. It can be inferred that the difference in hardness values in these three stages is due to the varying amounts of δ-phase precipitation.

For SLM alloys, the hardness values of the two cooling curves do not change significantly. This is because the δ-phase precipitates at the grain boundaries of SLM alloys, and some coarsening of the δ-phase occurs. During Stages I to III, the 0.5 °C/min cooling rate provides sufficient time for the strengthening phases to precipitate, resulting in a decrease in hardness. Therefore, the hardness curve of the 0.5 °C/min cooling rate is almost always below the FC curve, which is significantly different from the trend observed in DF alloys.

### 3.4. Microstructural Evolution During Non-Isothermal

#### 3.4.1. Microstructural Evolution During Heating

**Figure 8:**
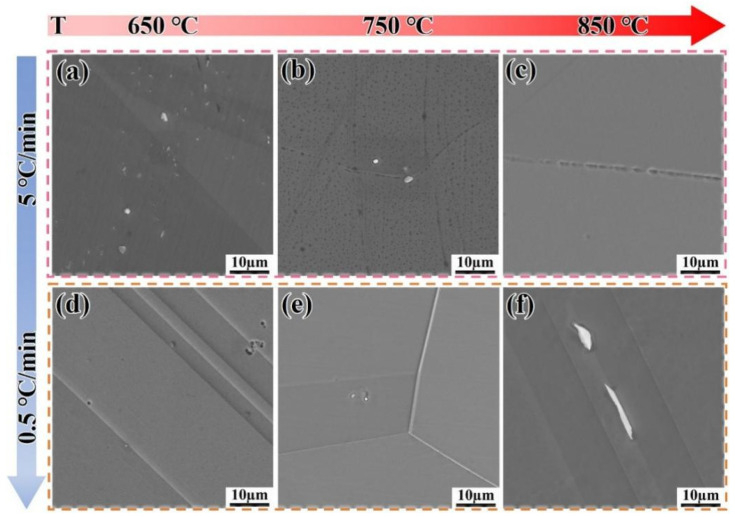
Microstructure evolution of the DF alloy heated to target temperatures at different heating rates. (**a**–**c**) 5 °C/min; (**d**–**f**) 0.5 °C/min.

Figure 8 shows the microstructure evolution of the DF alloy heated to 650 °C, 750 °C, and 850 °C at different heating rates. From Figure 8 it can be clearly seen that there is almost no precipitation phase at 650 °C and 750 °C, suggesting that the δ phase may have undergone re-dissolution. When heated to 850 °C at both heating rates, a certain degree of coarsening occurs at the grain boundaries (Figure 8c,f), leading to a significant decrease in hardness.

**Figure 9:**
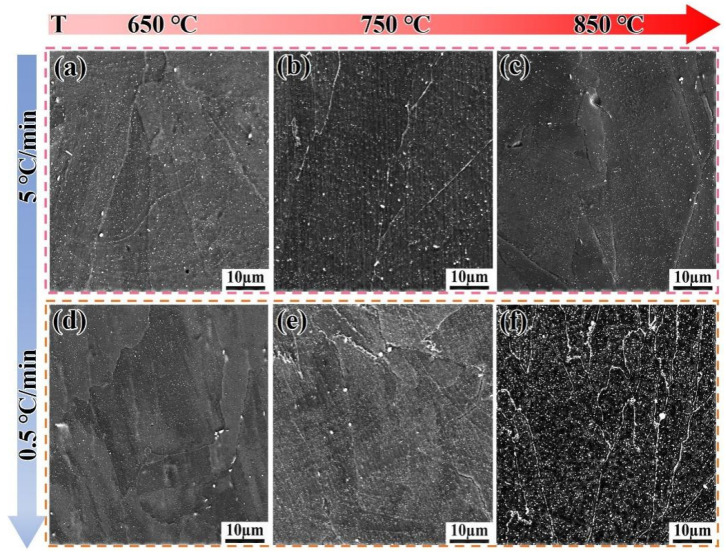
Microstructure evolution of the SLM alloy heated to target temperatures at different heating rates. (**a**–**c**) 5 °C/min; (**d**–**f**) 0.5 °C/min.

Figure 9 shows the microstructure evolution of the SLM alloy heated to 650 °C, 750 °C, and 850 °C at different heating rates. When heated at a rate of 5 °C/min, the volume fractions of the precipitated phases are 1.1%, 1.3%, and 1.5%, respectively, showing an increasing trend with temperature (Figure 9a–c). When the heating rate is reduced to 0.5 °C/min, the volume fractions of the precipitated phases are 2.1%, 2.9%, and 5.9%, respectively. This demonstrates that a slower heating rate provides sufficient time for the diffusion of Nb, Al, and Ti atoms, which is critical for the nucleation and growth of γ″ and γ′ phases [32]. The trend that slower heating rates promote a greater volume fraction of precipitates aligns well with the classic precipitation theory in nickel-based superalloys [32] and has been similarly observed in isothermal studies on wrought IN718 [33,34]. However, the present study quantifies this effect under non-isothermal conditions, revealing that the SLM alloy exhibits a more pronounced response to heating rate changes than the DF alloy, likely due to its finer initial microstructure [7,25]. The precipitated phases have sufficient time to form, and the precipitation rate accelerates without re-dissolution (Figure 9d–f). This is because, compared to the flat grain boundaries of the DF alloy, the twisted grain boundaries of the SLM alloy facilitate the precipitation of phases, preventing their re-dissolution.

**Figure 10:**
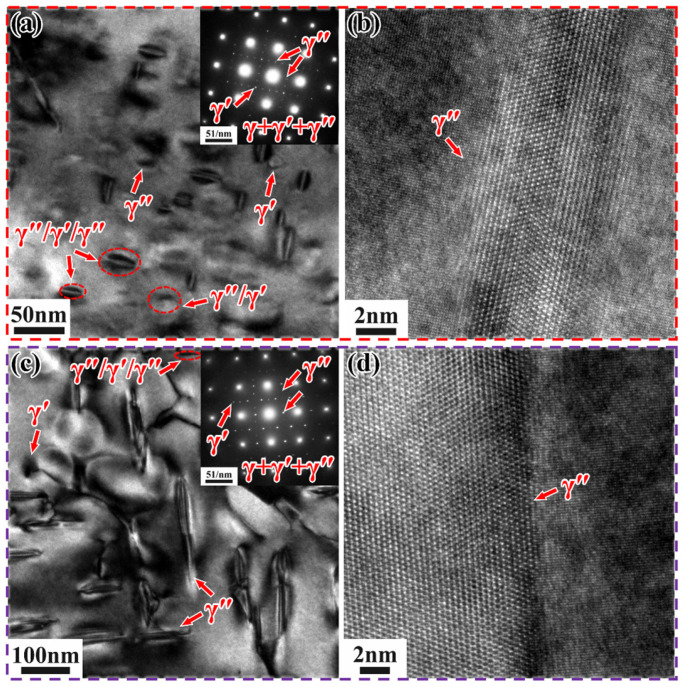
TEM images of Inconel 718 alloys heated to target temperatures at 0.5 °C/min. (**a**) Bright-field image and (**b**) HRTEM image of SLM alloy at 750 °C; (**c**) Bright-field image and (**d**) HRTEM image of DF alloy at 800 °C.

Figure 10 shows the TEM images of Inconel 718 alloys heated to 750 °C and 800 °C at 0.5 °C/min, along with the selected area electron diffraction (SAED) patterns in the upper right corner (along the [001] crystallographic zone axis). It is challenging to effectively quantify the γ″ and γ′ phases solely based on TEM and HRTEM images due to the low contrast between the γ′ and γ″ phases, making it difficult to distinguish them [35]. Therefore, in this study, only the total volume fraction of the precipitated phases was calculated.

In the SLM alloy, both γ′/γ″ bilayer precipitates and γ″/γ′/γ″ sandwich precipitates co-precipitate (Figure 10a). This is due to the co-precipitation of the γ′ and γ″ phases, resulting in the formation of composite γ′/γ″ structures [36,37]. However, the number of γ′/γ″ bilayer precipitates is extremely small compared to the γ″/γ′/γ″ sandwich precipitates, which are orthogonally distributed along two directions. These sandwich-like precipitates are characterized by γ″ precipitates sandwiched between γ′ precipitates, forming a γ″/γ′/γ″ structure. This structure results from the nucleation of γ″ precipitates from γ′ precipitates [34]. According to the (Al + Ti)/Nb atomic ratio, sandwich-like and dense precipitation types can be distinguished [33]. In this study, the (Al + Ti)/Nb atomic ratio is less than 0.9, leading to the formation of γ″/γ′/γ″ precipitates during non-isothermal aging heat treatment. These γ″/γ′/γ″ precipitates exhibit higher thermal stability compared to individual γ′ and γ″ precipitates [38]. The volume fraction of the precipitated phases is approximately 2.33%, with an average length and width of about 23.4 nm and 13.15 nm, respectively. It can be clearly observed that the γ″ phases form a good coherent relationship with the matrix (Figure 10b), which enhances the strengthening effect compared to incoherent reinforced phases [39]. In contrast, when the DF alloy is heated to 800 °C at 0.5 °C/min, a small amount of γ″/γ′/γ″ phase and γ′ phase appears, but γ″ phase is predominantly precipitated and orthogonally distributed (Figure 10c). The volume fraction of precipitates is approximately 5%, with an average length and width of about 138.4 nm and 24.7 nm, respectively. It can be clearly observed that the γ″ phase forms a good coherent relationship with the matrix (Figure 10d).

#### 3.4.2. Microstructural Evolution During Cooling

**Figure 11:**
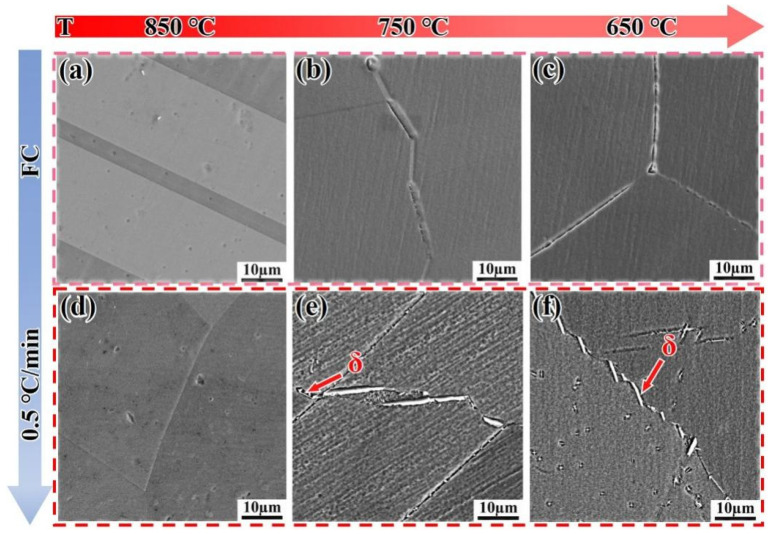
Microstructure evolution of the DF alloy cooled to target temperatures at different cooling rates. (**a**–**c**) Furnace cooling (FC); (**d**–**f**) 0.5 °C/mincooling.

**Figure 12:**
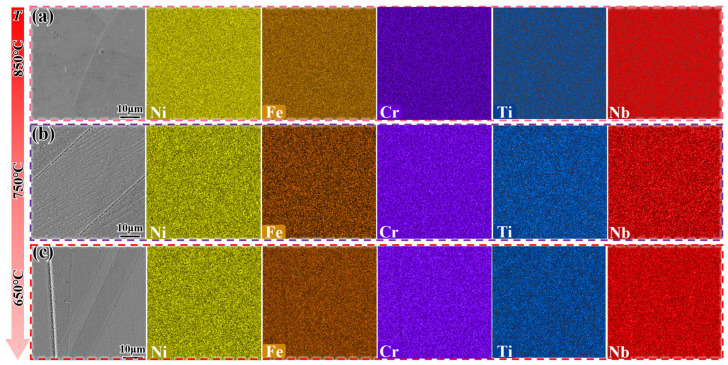
EDS analysis of the DF alloy cooled to different temperatures at 0.5 °C/min. (**a**) 850 °C; (**b**) 750 °C; (**c**) 650 °C.

Figure 11 shows the microstructure evolution of the DF alloy cooled to 850 °C, 750 °C, and 650 °C at different cooling rates. When the DF alloy is cooled by furnace cooling (FC), almost no precipitation phase is observed (Figure 11a–c). However, when the cooling rate is 0.5 °C/min, needle-like δ-phase precipitates appear, and their number increases as the temperature drops to 650 °C (Figure 11f). Figure 12 shows the EDS analysis of the DF alloy cooled to different temperatures at 0.5 °C/min. To verify the reason for the sudden drop in hardness within Stage II in Figure 7, the consumption of Nb elements needs to be confirmed. As the temperature decreases, Nb elements are released (as shown in Table 3), and a certain amount of Nb is retained in the early stages, causing the hardness value to exceed that of the FC samples within this stage.

**Figure 13:**
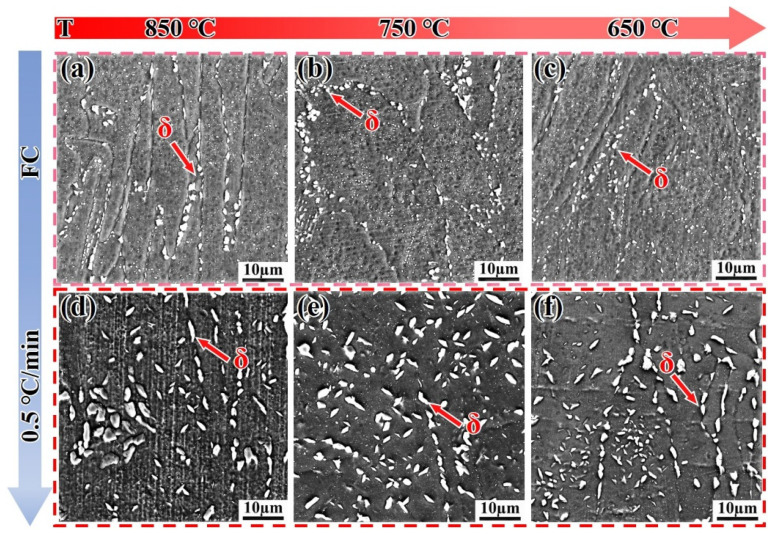
SEM images showing the microstructure evolution of the SLM alloy cooled to target temperatures at different cooling rates. (**a**–**c**) Furnace cooling (FC); (**d**–**f**) 0.5 °C/min cooling.

Figure 13 shows the microstructure evolution of the SLM alloy cooled to 850 °C, 750 °C, and 650 °C at different cooling rates. When the SLM alloy is cooled by furnace cooling (FC), the δ-phase is distributed along the grain boundaries in the form of granules (Figure 13a–c), with volume fractions of approximately 11%, 12.3%, and 10.7%, respectively, as the temperature decreases. The number of δ-phase precipitates shows a decreasing trend, and the hardness curves in Figure 7 exhibit small changes due to the coarsening of the δ-phase. Similarly, when the cooling rate is reduced to 0.5 °C/min, the δ-phase is still coarsened along the grain boundaries (Figure 13d–f), with volume fractions of 15.8%, 13.2%, and 12.1%, respectively. These values are larger compared to those of furnace cooling (FC), resulting in the hardness curve lying below the FC curve.

**Figure 14:**
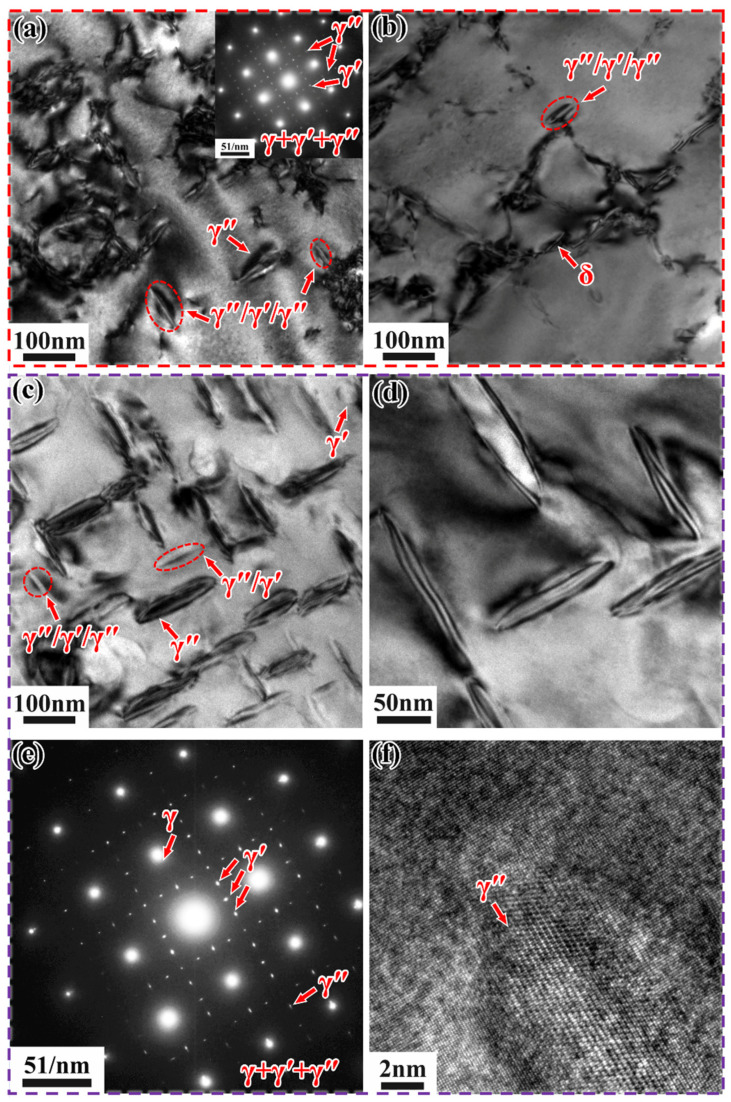
TEM microstructure of Inconel 718 alloys cooled to different temperatures at 0.5 °C/min. (**a**,**b**) Low-magnification bright-field images of SLM alloy at 600 °C; (**c**) Low-magnification bright-field image, (**d**) High-magnification bright-field image, (**e**) SAED pattern, and (**f**) HRTEM image of DF alloy at 700 °C.

Figure 14 shows the TEM microstructure of Inconel 718 alloys cooled to 600 °C and 700 °C at 0.5 °C/min, along with the corresponding bright-field images and selected area electron diffraction (SAED) patterns (along the [001] crystallographic zone axis).

When the SLM alloy is cooled to 600 °C at 0.5 °C/min, very few γ″/γ′/γ″ phases appear (Figure 14a,b), with an average length and width of approximately 108 nm and 37.1 nm, respectively. A small amount of needle-like δ-phase precipitates at the grain boundaries (Figure 14b), with an average length and width of 165 nm and 18.9 nm, respectively. The precipitation of the δ-phase consumes part of the Nb element, thereby suppressing the precipitation of the γ″/γ′/γ″ phase. When the DF alloy is cooled to 700 °C at 0.5 °C/min, a large number of strengthening phases precipitate from the matrix, and a small amount of γ′/γ″ phase and γ″/γ′/γ″ phase exists, with a total volume fraction of approximately 33%. These phases are orthogonally distributed in two directions (Figure 14c,d), with average lengths and widths of about 122 nm and 35.1 nm, respectively. This indirectly indicates that the cooling rate affects the quantity and distribution of the precipitated phases. It can be clearly observed that the γ″ phase forms a good coherent relationship with the matrix (Figure 14f).

### 3.5. Effect of Non-Isothermal Heat Treatment on Mechanical Properties

**Figure 15:**
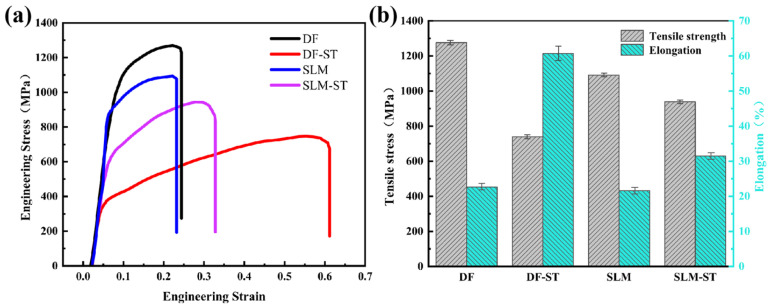
Tensile properties of Inconel 718 alloys prepared by traditional forging and 3D printing methods. (**a**) Engineering stress–strain curves; (**b**) Tensile strength and elongation corresponding to the tensile curves in (**a**).

Figure 15 presents the mechanical properties of Inconel 718 superalloys prepared by traditional forging and 3D printing methods, with detailed tensile strength and elongation values listed in Table 4. As can be seen from the figure and table, compared with the forged traditional Inconel 718 alloy samples, the deposited 3D-printed samples exhibit lower tensile strength and elongation.

By comparing the tensile results of Inconel 718 superalloys prepared by the two forming methods, this study finds that solution treatment has a greater impact on the mechanical properties of traditional alloy samples, but a smaller impact on those of 3D-printed alloy samples. After solution treatment, the tensile strength of the forged Inconel 718 alloy samples decreases by 537 MPa, with an overall reduction of 42.08%, while the elongation increases by 38.07%; for the deposited samples, the tensile strength decreases by 152 MPa, with an overall reduction of 13.93%, and the elongation increases by 9.83%. Therefore, compared with the 3D-printed Inconel 718 alloy samples, the tensile strength and elongation of the traditional Inconel 718 alloy samples undergo significant changes after solution treatment.

## 4. Conclusions

This work investigated the effects of continuous cooling and continuous heating treatments on the microstructural evolution and the resulting hardening and softening behaviors in additively manufactured and forged Inconel 718 alloys. The following conclusions can be drawn:

(1) The additively manufactured Inconel 718 alloy exhibited a unique microstructure, resulting in different heat treatment kinetics from those of forged alloys. Due to the fine dendritic structure, the additively manufactured alloy demonstrated a faster precipitation response than forged alloys, reaching peak hardness of ~480 HV at ~650 °C, which is approximately 100 °C earlier and 20 HV higher than the forged alloy’s peak hardness of ~460 HV at ~750 °C. The additively manufactured alloy reached peak hardness faster during heating and gained almost constant hardness during cooling.
(2) The heating rate significantly affected the precipitation behaviors of γ″ and γ′ phases in Inconel 718 alloy. Slower heating rates provided sufficient time for phase transformation, leading to a more pronounced precipitation. When the heating rate decreased from 5 °C/min to 0.5 °C/min, the volume fraction of precipitates in the SLM alloy increased from 1.5% to 5.9% at 850 °C. Both alloys achieved minimum hardness of ~380–390 HV at 1000 °C due to the full dissolution of γ″ and γ′ strengthening phases. The additively manufactured alloy exhibited accelerated aging precipitation behavior, with peak hardness occurring at lower temperatures compared to forged alloy.
(3) During cooling process, the hardness of additively manufactured Inconel 718 alloy showed minimal variation due to the precipitation and coarsening of δ-phase at twisted grain boundaries. In contrast, the flat grain boundaries of forged alloy led to the re-dissolution of δ-phase and a sudden increase in hardness due to the consumption of Nb.
(4) Comparison of tensile properties of DF (forged) and SLM (3D-printed) Inconel 718 alloys shows distinct effects of solution treatment: solution treatment has a far greater impact on the mechanical properties of DF alloy than on SLM alloy, which is linked to SLM’s unique microstructure and γ″/γ′ phase dissolution.

## Figures and Tables

**Table 1:** Chemical composition of forged and additively manufactured Inconel 718 alloys (wt.%).

Element	Ni	Cr	Fe	Nb	Ti	Mo	Co	Al
Forged	53.89	17.75	17.27	5.48	1.01	2.96	0.27	0.50
Additively manufactured	53.24	19.41	15.76	5.48	0.95	3.26	0.92	0.42

The chemical composition of both alloys was measured by Optical Analysis, which enables accurate quantitative detection of elemental content in metallic materials.

**Table 2:** Solidus and liquidus temperatures of Inconel 718 alloys.

Alloy	Solidus Temperature [°C]	Liquidus Temperature [°C]	Solidification Range [°C]
DF	1328	1348	20
SLM	1331	1348	17

**Table 3:** EDS results of the DF alloy cooled to different temperatures at 0.5 °C/min.

Temperature	Element Content [wt.%]				
	Fe	Cr	Nb	Ti	Ni
850 °C	16.98	18.08	4.7	0.93	Bal
750 °C	16.76	18	4.99	0.98	Bal
650 °C	16.65	17.71	5.07	0.91	Bal

**Table 4:** Tensile strength and elongation of Inconel 718 alloys prepared by traditional forging and 3D printing methods.

Materials	Tensile Strength [MPa]	Elongation [%]
DF	1276	22.62
DF-ST	739	60.09
SLM	1091	21.62
SLM-ST	939	31.45

## Data Availability

The original contributions presented in this study are included in the article. Further inquiries can be directed to the corresponding authors.
